# Pneumococcal LytR Protein Is Required for the Surface Attachment of Both Capsular Polysaccharide and Teichoic Acids: Essential for Pneumococcal Virulence

**DOI:** 10.3389/fmicb.2018.01199

**Published:** 2018-06-13

**Authors:** Weijie Ye, Jinghui Zhang, Zhaoche Shu, Yibing Yin, Xuemei Zhang, Kaifeng Wu

**Affiliations:** ^1^Key Laboratory of Diagnostic Medicine Designated by the Ministry of Education, Department of Laboratory Medicine, Chongqing Medical University, Chongqing, China; ^2^Department of Laboratory Medicine, The Third Affiliated Hospital of Zunyi Medical University, Zunyi, China

**Keywords:** *Streptococcus pneumoniae*, *lytR*, teichoic acids, capsular polysaccharide, virulence

## Abstract

The LytR-Cps-Psr family proteins are commonly present in Gram-positive bacteria, which have been shown to implicate in anchoring cell wall-related glycopolymers to the peptidoglycan. Here, we report the cellular function of SPD_1741 (LytR) in *Streptococcus pneumoniae* and its role in virulence of pneumococci. Pneumococcal Δ*lytR* mutants have been successfully constructed by replacing the *lytR* gene with *erm* cassette. The role of LytR in pneumococcal growth was determined by growth experiments, and surface accessibility of the LytR protein was analyzed using flow cytometry. Transmission electron microscopy (TEM) and immunoblotting were used to reveal the changes in capsular polysaccharide (CPS). Dot blot and ELISA were used to quantify the amount of teichoic acids (TAs). The contribution of LytR on bacterial virulence was assessed using *in vitro* phagocytosis assays and infection experiments. Compared to the wild-type strain, the Δ*lytR* mutant showed a defect in growth which merely grew to a maximal OD_620_ of 0.2 in the liquid medium. The growth of the Δ*lytR* mutant could be restored by addition of recombinant ΔTM-LytR protein in culture medium in a dose-dependent manner. TEM results showed that the D39Δ*lytR* mutant was impaired in the surface attachment of CPS. Deletion of *lytR* gene also impaired the retention of TAs on the surface of pneumococci. The reduction of CPS and TAs on the pneumocccal cells were confirmed using Dot blot and ELISA assays. Compared to wild-type D39, the Δ*lytR* mutant was more susceptible to the phagocytosis. Animal studies showed that the ability to colonize the nasophaynx and virulence of pneumococci were affected by impairment of the *lytR* gene. Collectively, these results suggest that pneumococcal LytR is involved in anchoring both the CPS and TAs to cell wall, which is important for virulence of pneumococci.

## Introduction

*Streptococcus pneumoniae* (*S. pneumoniae*) is a well-known human pathogen which, like other Gram-positive bacteria, is commonly decorated with teichoic acids (TAs) and capsular polysaccharide (CPS) on its outer surface of the bacterial body (Skov Sorensen et al., 1988; Hammerschmidt et al., 2005; Weidenmaier and Peschel, 2008). In *S. pneumoniae,* capsule is recognized as the most important determinant required for bacterial virulence, since less encapsulated or non-encapsulated pneumococcal strains are less virulent or avirulent (Hyams et al., 2010; Wu et al., 2016). TAs are another important glycopolymers which are comprised of cell-wall associated teichoic acid (WTA) and lipoteichoic acid (LTA) (Wu et al., 2014). TAs were implicated in several biological functions, including bacterial growth, bacterial division and morphology, and cellular roles in host-cell interaction (Wu et al., 2014; Liu et al., 2017).

The biosynthesis of both CPS and TAs is a complex process and requires multiple steps in *S. pneumoniae.* Except for serotypes 3 and 37, it has been indicated that the Wzy-dependent pathway was responsible for the biosynthesis of CPS (Arrecubieta et al., 1994; Llull et al., 1999). In addition, without counting the transcriptional regulators (Wu et al., 2016; Zheng et al., 2017), there are other proteins including Pgm and GalU which are involving in sugar metabolism determine the CPS biosynthesis (Mollerach et al., 1998; Hardy et al., 2001). As for the biosynthesis of TAs, a previous bioinformatic analysis suggested the involvement of 16 known genes and other hypothetical genes, primarily clustered in the *lic* loci, in the biosynthesis of pneumococcal TAs (Denapaite et al., 2012). In 2014, we found that RafX was implicated in the ligation of WTA to the cell wall (Wu et al., 2010). Recent study by Liu et al. has revealed that SPD_1198 and SPD_1197 were responsible for the polymerization process of TAs (Liu et al., 2017). Despite current progress in identification of these enzymes, so far, not all the critical enzymes involved in the biosynthesis of both glycopolymers are identified.

Previous work by Kawai et al. has suggested the function of LytR-CpsA-Psr family proteins (LCP) in the attachment of WTA to peptidoglycan in *Bacillus subtilis (B. subtilis)* (Kawai et al., 2011). Other studies in *Staphylococcus aureus* (*S. aureus*) and *B. anthracis* have also revealed the necessary of LCP enzymes in the attachment of either CPS or TAs to the cell wall (Chan et al., 2013, 2014; Liszewski Zilla et al., 2015). More recently, Gale et al. (2017) and Schaefer et al. (2017) using *in vitro* enzymatic assays demonstrated independently the ligase activities of LCP in attaching the WTA to the peptidoglycan in *S. aureus* and *B. subtilis* . These lines of evidence indicated that LCP proteins have ligase activity which contributed to the attachment of WTA to the cell wall and probably the CPS to the cell wall.

In *S. pneumoniae*, there are three proteins including SPD_1741 (LytR), Cps2A, and SPD_1202 (Psr) belonging to the LCP family. It has been indicated that Cps2A has the phosphotransferase activity and knocking down *cps2A* reduced the amount of capsule on the cell wall (Eberhardt et al., 2012). Inactivation of both *cps2A* and *lytR* (*SPD_1741*) significantly impaired the retention of CPS on the cell wall in *S. pneumoniae* D39 (Eberhardt et al., 2012). It is known that knocking down *cps2A* alone is sufficient to cause obvious loss of CPS, speculating the involvement of LytR in CPS production is indirect and may be not sufficient. While two previous studies have showed that pneumococcal LytR is essential for *S. pneumoniae* viability (Johnsborg and Havarstein, 2009; Eberhardt et al., 2012), using the CRISPRi knockdown technique, Liu *et al.* did not prove the *lytR* as the necessary gene in *S. pneumoniae* (Liu et al., 2017). These motivate us to further clarify the function of the LytR in S. *pneumoniae* by preparing single *lytR* mutant.

In this study, pneumococcal *lytR* gene was successfully replaced with *erm* cassette to obtain *lytR* mutants. We found that the Δ*lytR* mutant was impaired in growth, and addition of recombinant LytR protein to the culture medium restored the growth in a dose dependent manner. Using TEM analysis and other immunological analytical methods, we showed that pneumococcal LytR was involved in both the attachment of CPS and TAs to the cell wall. Finally, we showed that the inactivation of LytR may be disastrous to *S. pneumoniae* during infecting the host.

## Materials and Methods

### Bacterial Strains, Plasmids, Bacterial Growth Conditions

All of the bacterial strains, plasmids, and primers used in this study are listed in Supplementary Tables S1, S2 in Supplementary Material. *E. coli* strains were grown in Luria-Bertani (LB) broth with shaking or on LB agar plates at 37°C. *S. pneumoniae* strains were grown in semisynthetic casein hydrolysate medium supplemented with 5% yeast extract (C+Y medium, pH 7.0) or on blood agar plates at 37°C in an atmosphere of 5% CO_2_. Bacteria were stored in growth medium supplemented with 10% glycerol at -80°C. Appropriate antibiotics (Supplementary Table S1) were supplemented to bacterial growth medium when needed.

### Expression and Purification of the ΔTM-LytR Protein

Since the first 30 amino acid residues belong to the trans-membrane domain (TM), to facilitate prokaryotic expression of soluble protein, the ΔTM-LytR encoding region was amplified from the genome of *S. pneumoniae* D39 using PCR technology with primer pairs LytR-F and LytR-R (Supplementary Table S2). The resulting *lytR* product was purified and digested with restriction enzymes, and then cloned into the pW28 plasmid which was further used to transform *E. coli BL21* (DE3) competent cell. The *E. coli* bacteria with ΔTM-LytR-pW28 plasmid were grown in 500 ml LB medium supplemented with 50 μg/ml kanamycine for 8 h in a shaking incubator. Isopropyl β-D-Thiogalactoside (IPTG) at a concentration of 0.25 mM was used to induce the expression of rΔTM-LytR protein. Bacteria were pelleted at 6000 rpm for 30 min at 4°C, resuspended in 25 ml binding buffer containing 100 μM PMSF, and then were subjected to sonication. rΔTM-LytR protein was purified using Ni^2+^-NTA sepharose column, its purity was analyzed by SDS-PAGE. Aliquots of rΔTM-LytR protein were stored at -80°C.

### Construction of *lytR* Mutants

The primers used in this study were listed in Supplementary Table S2. A length of 2467-bp cassette containing *SPD_1741* (*lytR*) upstream and downstream fragments and *erm* encoding sequence was generated by PCR using the primers ΔlytR-P1, ΔlytR-P2, ΔlytR-P3, and ΔlytR-P4. The resulting fragment was used to transform the *S. pneumoniae* strains. The transformation procedures, colony selection and identification were performed according to established protocols (Wu et al., 2010). Erythromycin resistant colonies were selected and the targeted sequences were amplified by PCR and sequenced.

### Construction of Complemented Strains

The plasmid pJWV25 with full-length of *lytR* gene was used to complement the Δ*lytR* mutants (Eberhardt et al., 2009). The full-length *lytR* fragment was cloned into plasmid pJWV25 at the *Spe* I and *Not* I restriction sites. The resulting recombinant plasmid was used to transform Δ*lytR* mutants and selected by tetracycline to obtain the complemented strains (Δ*lytR*-C). For functional assays, the complemented strain was grown in normal C+Y medium or supplemented with 0.15 mM Zn^2+^.

### Growth Curve

A volume of 10 μl of stored wild-type D39 strain and the D39Δ*lytR* mutant were grown on Columbia blood agar plates for 16 h. Bacteria were harvested from the blood agar plates and washed twice in phosphate-buffered saline (PBS). Bacteria were resuspended to an OD_620_ = 0.4. For growth on Columbia blood agar plate, 10 μl of bacterial suspensions of wild-type D39 and the D39Δ*lyt*R mutant were separately inoculated onto one Columbia blood agar plate and grown at 37°C in an atmosphere of 5% CO_2_ for 8 h. For growth experiment in liquid medium, suspensions of 100 μl were seeded into 5 ml fresh liquid medium. The wild type D39 and the Δ*lytR* mutant were grown in normal C+Y medium. D39Δ*lyt*R mutant was grown in C+Y medium with different amounts of rΔTM-LytR (1 ng, 10 ng, 100 ng and 500 ng, respectively) to evaluate the role of LytR in bacterial growth. D39Δ*lytR* grown in C+Y medium with 500 ng pneumolysin (PLY) was used as a negative control. Bacteria were cultured at 37°C in an atmosphere of 5% CO_2_, and OD_620_ values were recorded at the indicated times.

### Transmission Electron Microscopy (TEM)

A volume of 10 μl of stored wild-type D39 strain and the D39Δ*lytR* mutant were grown on Columbia blood agar plates for 16 h. Bacteria were harvested from the blood agar plates and washed twice in phosphate-buffered saline (PBS). Bacteria were resuspended to an OD_620_ = 0.4. A volume of 200 μl of bacterial suspension was added to 10 ml fresh C+Y medium or C+Y medium supplemented with 0.15 mM ZnCl_2_ (Δ*lytR-*C) and cultured at 37°C till an OD_620_ = 0.15 for the *lytR* mutant and an OD_620_ = 0.4 for the others. Bacteria were harvested by centrifugation at 10 000 rpm for 20 min at 4°C. Samples were fixed in 1 ml sodium cacodylate buffer supplemented with 2% glutaraldehyde (pH7.4) at 4°C for 16 h before being subjected to analysis. Other procedures were processed by the Electron Microscopy Research service of Chongqing Medical University.

### Analysis of TAs/CPS

Bacteria were prepared as described for TEM analysis. Bacteria were harvested by centrifugation at 10 000 rpm for 5 min. The cells were washed twice in phosphate-buffered saline (PBS), and lysed with 0.5% sodium deoxycholate solution (DOC) containing PMSF at 37°C for 30 min. Cells were observed by optical microscope to ensure complete lysis. Protein levels were determined by Bradford method (Bio-Rad Protein Assay). For standardization, the protein levels in all bacterial lysates were adjusted to a concentration of 4 mg/ml, and the protein levels were adjusted to concentrations of 1 mg/ml and 3 mg/ml for culture supernatant of R6 and D39, respectively. The amounts of CPS and TAs were analyzed by Western blot, Dot blot, or enzyme-linked immunosorbent assay (ELISA) according to the established protocols (Wu et al., 2014; Zheng et al., 2017).

### Fluorescence Activating Cell Sorter (FACS)

Bacteria were prepared as described above. The bacterial cells were harvested by centrifugation at 12,000 rpm for 5 min and washed twice in PBS. 1 × 10^6^ CFU bacteria were inactivated in water bath at 56°C for 30 min, resuspended in 200 μl PBS containing 1 μl anti-CWPS or anti-LytR, and incubated at 37°C for 60 min. Cells were washed twice with PBS, and probed with 200 μl PE-labeled goat anti-rabbit IgG or goat anti-mouse IgA (diluted at 1:200, secondary antibodies). After three washes with PBS, cells were subjected to detection. Samples were analyzed by technicians in Children’s Hospital of Chongqing Medical University.

### Animal Studies

6–8-week-old C57BL/6 mice were obtained and raised at the experimental animal center, Chongqing Medical University [certificate no. SYXK(yu) 2007-0001]. All the animal experiments were discussed with and approved by the Animal Care and Use Committee of Chongqing Medical University. All procedures were performed according to the recommendations in the Guide for the Care and Use of Laboratory Animals, and conformed to animal protection laws of China and applicable guidelines.

Bacteria were prepared as described above. The Δ*lytR*-C strain used in animal experiments was grown in C+Y supplemented with 0.15 mM ZnCl_2_ to induce the expression of LytR. Groups of 6-week-old male C57BL/6 mice were intranasally challenged with 1 × 10^7^ CFU of bacteria. Survival of the animals was recorded every day until the end of the observation period (15 days). As for colonization experiments, mice were sacrificed after infection with pneumococci for 24 h or 48 h. Bacteria was harvested from nasal lavage fluid which was serially diluted and plated on the blood agar plates to have bacterial numbers.

Lungs were inflated with 0.5% agarose under 25-cm water pressure and fixed in 10% buffered formalin for 24 hours. Next, formalin-fixed, paraffin-embedded 4-mm sections of lungs were used for immunohistochemical analysis with hematoxylin eosinstain (Hoogerwerf et al., 2011; Cao et al., 2014).

### Anti-Phagocytosis Assays

Bacteria were prepared as described above. Density of bacterial suspension was adjusted to OD_620_ = 0.4. 200 μl of bacterial suspension was added to 10 ml fresh C+Y medium. The wild-type D39 and Δ*lytR-*C and the Δ*lytR* grown with *LytR* protein were cultured to mid-exponential phase (OD_620_ = 0.4). The Δ*lytR* mutant and Δ*lytR+*PLY protein were cultured to an OD_620_ = 0.15. The bacterial density was adjusted to 1 × 10^7^ CFU/ml. Macrophages (1 × 10^5^ cells) derived from mice peritoneal cavity were incubated with bacteria at a MOI 100 at 37°C for 30 min according to previous protocols with modifications (Kodali et al., 2013; Sharif et al., 2013). After three washes with PBS, cells were lysed with ddH_2_O and plated onto blood agar plates. The bacteria numbers were counted and survival rates were calculated.

### Statistical Analysis

Differences between groups were analyzed by two-tailed *t*-test or Mann–Whitney U test using Graph-Pad Prism 5 software. Survival rates were compared using the log-rank (Mantel–Cox) test. A *P* value of ≤ 0.05 was considered statistically significant.

## Results

### Expression and Purification of ΔTM-LytR Protein

**FIGURE 1:**
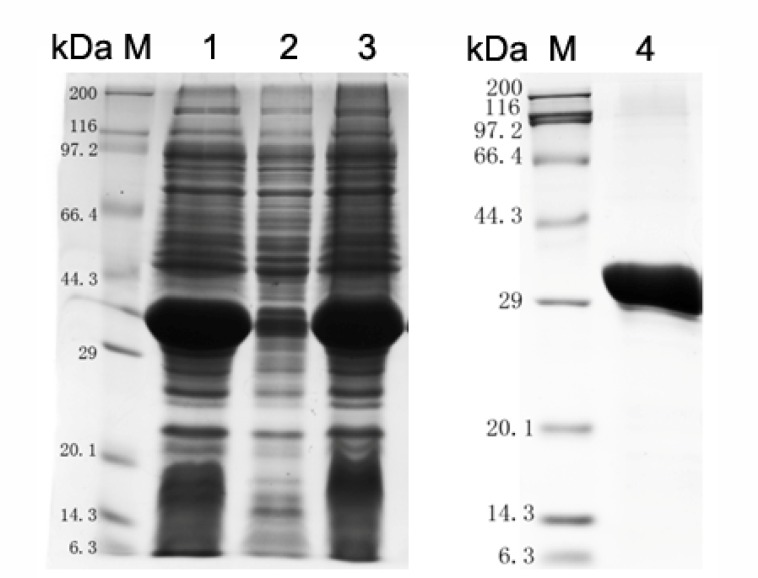
Analysis of LytR protein expression by SDS-PAGE. M: Protein marker; lane 1: whole cell lysate; lane 2: inclusion body; lane 3: soluble form; lane 4: purified ΔTM-LytR. The purified ΔTM-LytR migrated at the predicted molecular mass of ∼35 kDa.

Using online bioinformatic tools, it was found that *S. pneumoniae* SPD_1741 (LytR) protein was a hydrophilic protein with a relative molecular weight of 37.56 kDa and an iso-electric point of 5.11. *S. pneumoniae* LytR protein belongs to the LytR_cpsA_psr family and was predicted to be an anionic cell wall polymer synthesis related enzyme. Our attempts to prepare full length LytR protein were unsuccessful. By structural analysis using TMHMM online software (Supplementary Figure S1), it was found that LytR protein contains 1 predicted transmembrane (TM) helices within the first 30 amino acid residues at the N-terminus, and the enzymatic domain was located on the outside the cell membrane. Therefore, only the predicted extracellular domain of LytR protein was overexpressed in *E. coli* BL21 (DE3). As shown in **Figure 1**, the recombinant ΔTM-LytR could be generated in soluble forms. Over 90% purity could be achieved by purification with the Ni^2+^-NTA affinity chromatography. The purified rΔTM-LytR protein was used to prepare the anti-LytR antiserum and used for following functional assays.

### Surface Accessibility of LytR Protein in *S. pneumoniae*

**FIGURE 2:**
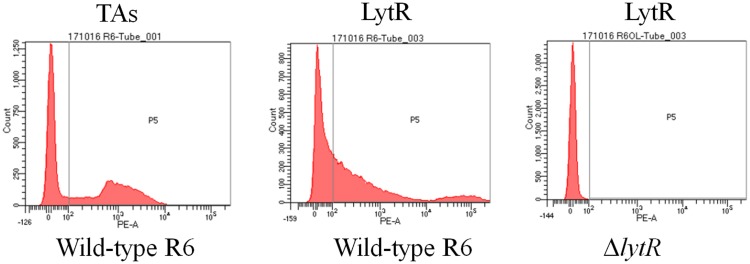
Surface accessibility of pneumococcal LytR. Wild-type R6 (WT) was incubated with either anti-CWPS antiserum (probes TAs) or anti-LytR antiserum and probed with the PE-labeled secondary antibodies. Δ*lytR* mutant was used as negative control. Three independent experiments were performed and representative figures were presented.

TMHMM analysis showed that the enzymatic domain of LytR was located outside the cell membrane (Supplementary Figure S1). In addition, tracing with GFP protein, it has been shown that LytR was located in the membrane (Eberhardt et al., 2012). However, it remains unclear whether the enzymatic domain of LytR was exposed outside the bacterial bodies. It has been shown that LytR was expressed throughout the growth phase (Johnsborg and Havarstein, 2009). Therefore, pneumococci grown in the early exponential phase were collected for FACS analysis. Wild-type R6 and Δ*lytR* mutant were incubated with anti-LytR antiserum, and probed with PE-labeled goat anti-mouse secondary antibodies. The surface location of TAs was used as positive control. As shown in **Figure 2**, it was found that, compared to the Δ*lytR* mutant showing no positive fluorescence signal, the LytR signal in wild-type R6 was obvious. Combined with evidence available, it was strongly suggested that LytR is a membrane protein and its predicted enzymatic domain is surface accessible.

### LytR Protein Was Essential for Pneumococcal Growth

To test the function of *S. pneumoniae* LytR, we created the Δ*lytR* mutant on the genetic background of R6 and D39 in which the *lytR* gene was replaced with *erm* cassette using insertion-deletion mutagenesis strategy. The deletion of *lytR* gene was proved to be a nonpolar event verified by reverse transcription (RT)-PCR. To further confirm the role of LytR, we complemented the *lytR* gene ectopically with the pJWV25 plasmid. Sequencing of the respective genome regions confirmed that the mutations or insertions generated as expected.

**FIGURE 3:**
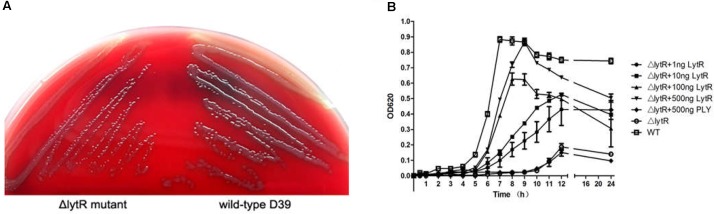
Growth characteristics of the strains. Equal volumes containing equal bacterial numbers of wild-type D39 and the Δ*lytR* mutant were grown on blood agar plates for 8 h **(A)**. Growth curves of strains grown in C+Y medium or medium supplemented with the indicated proteins **(B)**. Cell densities were measured at the indicated time points. The control protein, PLY, not involved in bacterial control was used as control. The values represent means of three independent with SEM.

Inactivation of *lytR* led to significant growth defects in either the blood agar plate or the liquid medium. Compared with wild-type D39, the Δ*lytR* mutant was smaller but remained smooth like wild-type D39 (**Figure 3A**). When growing in the liquid medium, the Δ*lytR* mutant showed significantly reduced growth rate compared to the wild-type strain. The D39Δ*lytR* mutant only grew to a maximal OD_620_ of 0.2 (**Figure 3B**).

As shown in **Figure 2**, since LytR in wild type pneumococci was surface exposed, we wanted to see whether the addition of the recombinant ΔTM-LytR could complement the growth defect. 1 ng, 10 ng, 100 ng, and 500 ng recombinant ΔTM-LytR proteins were added into the culture medium of Δ*lytR* mutant and the optical densities were measured at OD_620_, respectively. As a control, 500 ng of the recombinant pneumolysin (Ply) was added into the culture medium of the Δ*lytR* mutant. As shown in **Figure 3B**, the recombinant ΔTM-LytR improved growth of the Δ*lytR* mutant in a dose-dependent manner, while the addition of Ply was ineffective.

### LytR Was Required for Attachment of the Capsular Polysaccharide to Cell Wall

**FIGURE 4:**
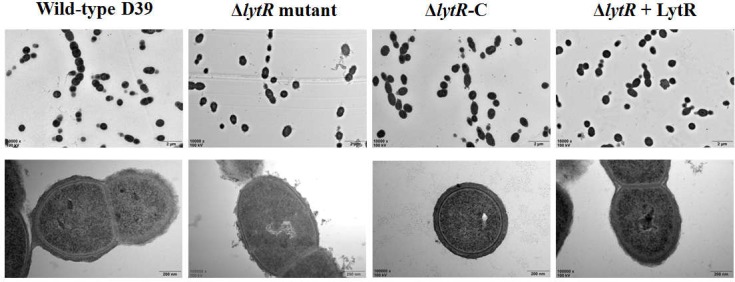
Electron micrographs of strains. Representative figures are shown. Δ*lytR* mutant: D39Δ*lytR*; Δ*lytR*-C: complemented strain; Δ*lytR* + LytR: mutant grown in the presence of 500 ng ΔTM-LytR protein.

Since the Δ*lytR* mutant grown on blood agar plate displayed small and smooth colony phenotypes, and considering previous indication regarding the role of LCP family proteins in the biosynthesis of glycopolymers, we analyzed the characteristics of the *lytR* mutant by TEM (**Figure 4**). The complemented *lytR* mutant and the Δ*lytR* mutant grown in the medium supplemented with 500 ng recombinant ΔTM-LytR protein were analyzed in parallel. The TEM results showed that the thickness of capsule present on the bacterial surface was significantly reduced in D39Δ*lytR* mutant cells relative to that of the wild-type D39 cells, indicating a defect in CPS biosynthesis. Both the complemented strain (Δ*lytR*-C) and the Δ*lytR* mutant grown in the surrounding with recombinant LytR protein restored the capsule, suggesting the direct involvement LytR in attaching the CPS to the cell wall.

**FIGURE 5:**
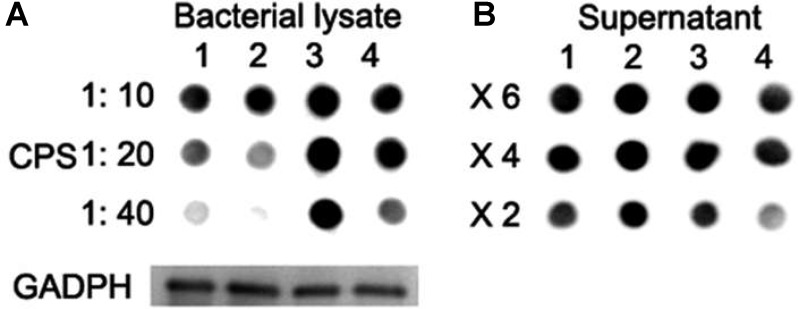
CPS quantification with Dot blot assays. **(A)** Lane 1: wild-type D39; lane 2: Δ*lytR* mutant; lane 3: complemented strain; lane 4: Δ*lytR* mutant grown in C+Y medium plus 500 ng LytR protein. 1:10, 1:20 and 1:40 indicate the diluted fold of samples. **(B)** X6, X4 and X2 indicate the concentration fold of the culture medium. GADPH was used as loading control. Three independent experiments were performed and representative figures were presented.

Immnoblotting assays were further performed to determine the production of CPS in lysates of strains. Wild-type D39 and D39Δ*lytR* strains were grown in normal C+Y medium. For the complemented strain, cells were grown in C+Y medium supplemented with 0.15 mM ZnCl_2_. Bacteria were harvested, re-suspended in lysis buffer, and then subjected to immunoblotting assays using rabbit anti-serotype 2 antiserum. GADPH was used as an internal control. Dot blot results showed that, compared with wild-type D39 strain, D39Δ*lytR* mutant was reduced in the amount of CPS in the bacterial lysates (**Figure 5A**). Surprisingly, more CPS in the bacterial lysates was observed in complemented strain and Δ*lytR* mutant supplemented with LytR protein in comparison with the wild-type strain. We also analyzed the amount of CPS in the culture medium, and it was found that more CPS could be detected in the culture supernatant of the Δ*lytR* mutant (**Figure 5B**). These results confirmed the results from TEM analysis, and further suggested the direct involvement of LytR in anchoring the CPS to the cell wall.

### LytR Was Involved in the Attachment of Pneumococcal TAs

**FIGURE 6:**
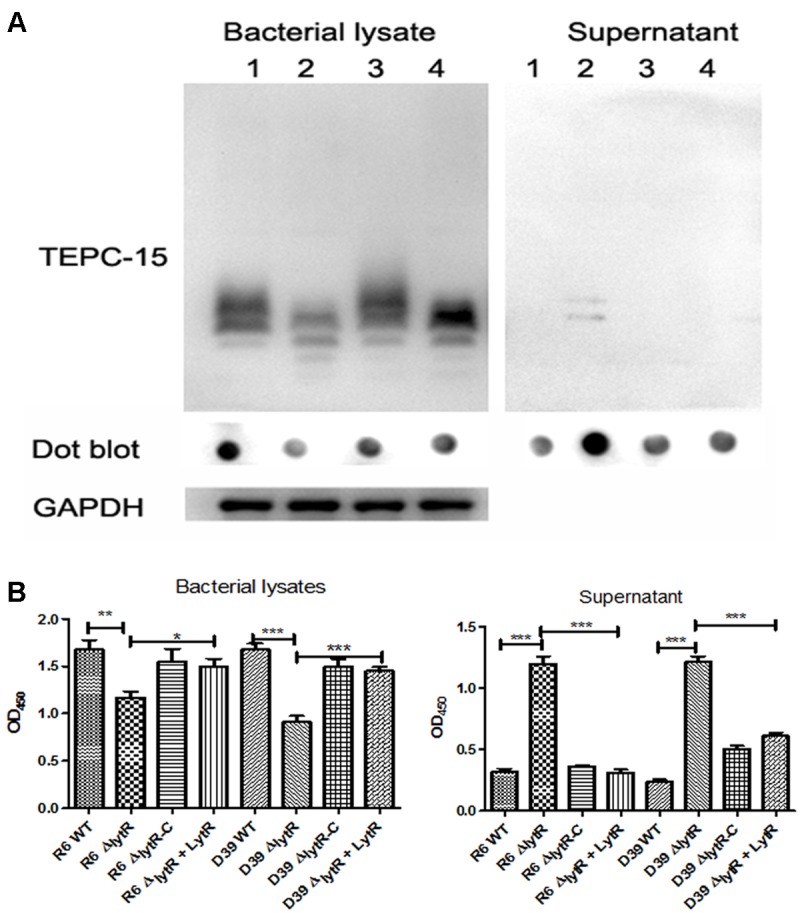
The LytR contributes to TA biosynthesis in *S. pneumoniae.* **(A)** Teichoic acids were analyzed using Western blot and Dot blot, in which TEPC-15 was used to probe the TAs. GADPH was used to indicate the equivalent of the sample loading. Lane 1: wild-type D39; lane 2: *lytR* mutant; lane 3: complemented strain; lane 4: *lytR* mutant grown in C+Y medium plus LytR protein. Three independent experiments were performed and representative figures were presented. **(B)** TAs in bacterial lysates and supernatant were determined using ELISA. Three independent experiments were performed and the data represent means of three replicates ± standard deviations. For bacterial lysates, R6 WT *vs* R6Δ*lytR, P* = 0.0061; R6Δ*lytR vs* R6Δ*lytR*-C, *P* = 0.02. ^∗^*P* < 0.05, ^∗∗^*P* < 0.01, ^∗∗∗^*P* < 0.0001.

It has been indicated that the pneumococcal TAs were related with the growth of pneumococci (Liu et al., 2017). As shown in **Figure 3B**, the Δ*lytR* mutant was impaired in growth. We therefore hypothesized that the *lytR* gene may be involved in the attachment of TAs to bacterial surface. Western blot was used to analyze the amount of TAs in whole-cell lysates using monoclonal antibody against phosphorylcholine of TAs (TEPC-15). Wild-type D39 and the Δ*lytR* mutant were grown in normal C+Y medium. Cells of complemented strain were grown in the presence of 0.15 mM ZnCl_2_. The amounts of TAs in bacterial lysates and supernatant of the Δ*lytR* mutant grown in the C+Y medium supplemented with 500 ng LytR were also determined. Whole-cell lysates were separated with SDS-PAGE, and TAs were probed with TEPC-15. As shown in **Figure 6A**, compared to wild-type strain, the Δ*lytR* mutant showed smaller TA bands and the density of the bands is weaker. We hypothesized that TAs were released into the culture medium. We therefore tested the amounts of TAs in the culture supernatant using Western blot and Dot blot. It was found that, compared to wild-type and the complemented strains, more TAs in the culture medium of the Δ*lytR* mutant could be detected. To confirm the changes observed in Western blot assays, the amount of TAs was determined by ELISA. In line with the Western blot results, the amounts of TAs in the *lytR* mutants were about 2/3 of the levels of the wild-type D39 (**Figure 6B**), and significant more TAs were detected in the culture medium of the *lytR* mutant. Complementation with LytR restored the amount of TAs.

### Role of LytR in Resistance to Phagocytosis

**FIGURE 7:**
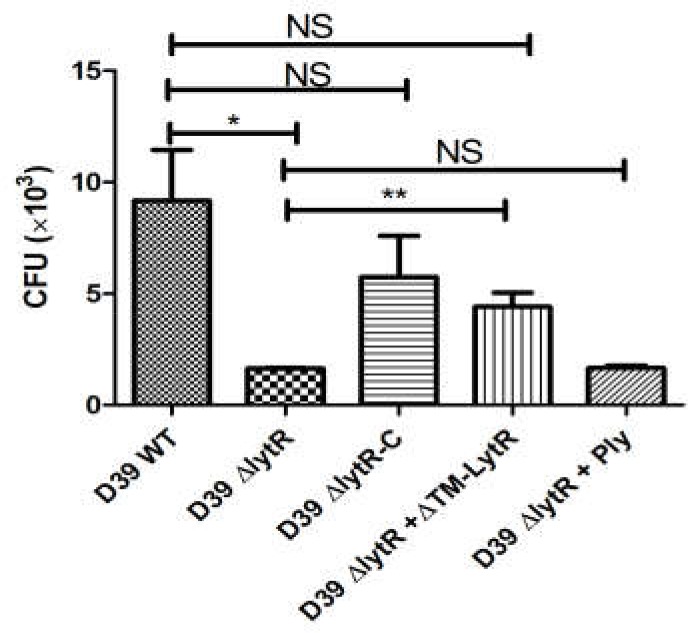
Pneumococcal LytR contributed to resistance to phagocytosis. D39Δ*lytR* + ΔTM-LytR: D39Δ*lytR* cells were grown in the presence of 500 ng LytR; D39Δ*lytR* + Ply: D39Δ*lytR* cells were grown in the presence of 500 ng Ply. D39 WT *vs* D39Δ*lytR*, *P* = 0.016; D39Δ*lytR* + LytR *vs* D39Δ*lytR, P* = 0.0052; ^∗^*P* < 0.05, ^∗∗^*P* < 0.01; NS: not significant. Three independent experiments were performed and the data represent means of three replicates ± standard deviations.

Given the critical role of LytR in biosynthesis of both CPS and TAs, we next tested the impairment of LytR mutation in bacterial virulence using *in vitro* anti-phagocytosis assays. Wild-type D39, the *lytR* mutant, complemented strains were incubated with macrohages cells for 30 min, and cell lysates were plated onto the blood agar plates for bacterial counting. As shown in **Figure 7**, the *lytR* mutant was very sensitive to the killing by macrophages, showing five- to sixfold reduction in survival of the *lytR* mutant in relative to that of the wild-type strain (**Figure 7**). The addition of Ply in the culture medium of the *lytR* mutant failed to improve the survival, while the addition of LytR significantly improved the survival of the *lytR* mutant. This observation suggested that the LytR protein is critical for the full virulence of pneumococci.

### LytR Mutation Attenuated the Pneumococcal Virulence

**FIGURE 8:**
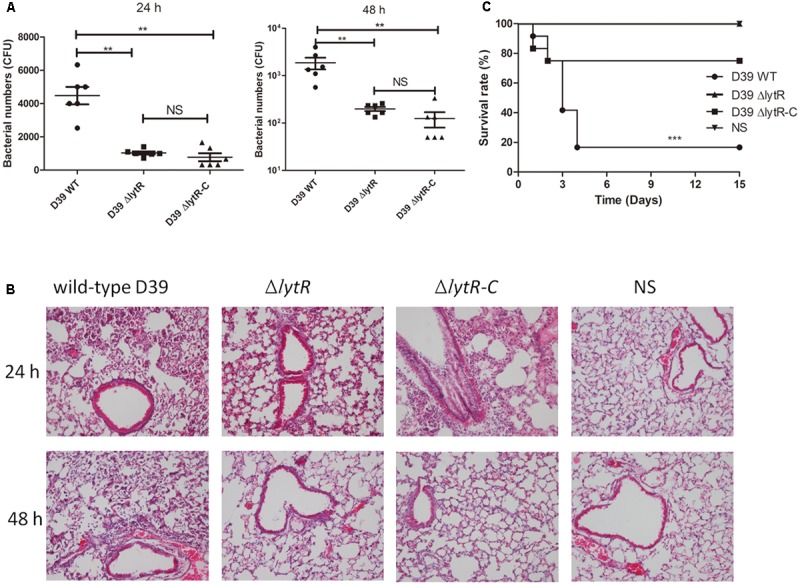
The role of LytR in nasopharyngeal colonization and systemic virulence. **(A)** C57BL6 mice were challenged with 10^7^ CFU *S. pneumoniae* D39 and pneumococci were recovered from the nasopharynx 24 or 48 h after intranasal infection of the mice. The graphs show bacterial numbers (CFUs) recovered from nasopharynx of six mice. Data are the mean (SD). ^∗∗^*P* < 0.01; Statistical deference was determined by unpaired two-tailed Student’s *t*-test. **(B)** HE staining images of lung tissues of mice 24 h and 48 h after challenge with the indicated strains with the same challenge dose as the colonization experiments. Δ*lytR*-C: complemented strain. NS: Normal saline water. **(C)** C57BL6 mice were infected intranasally with 10^7^ cells of bacteria. Survival of the mice was recorded, and the difference in survival rates was analyzed using log-rank. ^∗∗∗^*P* < 0.0001.

To further understand the role of LytR in pneumococal virulence, we assess the contribution of LytR in pneumococcal colonization and systemic virulence. To determine whether LytR contribute to pneumococcal colonization at the respiratory tracts, groups of twelve C57BL6 mice were inoculated intranasally with wild-type D39, D39Δ*lytR*, or Δ*lytR*-C at a dose of 10^7^ CFU. Six mice from each group were killed after 24 and 48 h, and the numbers of bacteria recovered from nasopharynx were determined and compared (**Figure 8A**). Compared to wild-type D39 strain, the Δ*lytR* mutant showed a significant decrease in numbers of pneumococci colonizing the nasopharynx at both 24 h and 48 h. Compared with the wild-type D39 strain, a significant decrease in bacterial load of the nasopharynx was also observed at both 24 h and 48 h postinoculation for the Δ*lytR*-C strain.

Furthermore, 24 h and 48 h after intranasal infection with *S. pneumoniae* D39, lung tissues were removed and subjected to histological examination using hematoxylineeosin staining (**Figure 8B**). The results showed that, mice challenged with the *lytR* mutant only had a mild inflammation in their lungs. Inflammation was readily apparent in mice challenged with the wild-type D39, with obvious infiltration of neutrophils and monocytes, and even severe necrotic debris in lungs. Inflammation was apparent in mice challenged with the complemented strain at 24 h, but was readily absorbed at 48 h. Obvious inflammation was not observed in mice challenged with normal saline water (NS) at both 24 h and 48 h.

To investigate the role of LytR in systemic infection, groups of twelve C57BL6 mice were challenged intranasally with wild-type D39, D39Δ*lytR* or Δ*lytR*-C at a dose of 10^7^ CFU. Mice receiving NS was used as negative control. As shown in **Figure 8C**, all mice challenged with D39Δ*lytR* and NS survived at the end of the observation period, and 83.3% (10/12) of the mice died from the infection with wild-type D39. The differences in survival rate were significant among the groups, suggesting that LytR is important for full virulence of pneumococci.

## Discussion

Here, by inactivating *lytR* gene alone, we present evidence that the *S. pneumoniae* LytR protein is essential for growth and we suggest that it is responsible for attachment of both CPS and TAs to the cell wall. In addition, in line with its function, we observed a significant reduction in pneumococcal virulence when LytR was inactivated. Thus, the present study increases our knowledge concerning the biosynthesis of pneumococcal cell wall.

Gale et al. (2017) has reported that *B. subtilis* LCP proteins, by acting as a ligase, attached WTAs to peptidoglycan *in vitro* . By comparing the sequence identity, it was found that *S. pneumoniae* LytR (SPD_1741) showed 32.5% identity with the *B. subtilis* DJ97_1211 in full-length amino acid sequence (Supplementary Figure S2). In this study, it was impressive that the D39Δ*lytR* mutant was impaired in both the retention of CPS and TAs. Exogenous addition of LytR protein restored the attachment of both CPS and TAs to the bacterial cells. In *Staphylococcus aureus*, the phosphosugar transferase activity of LCP proteins has been presented by showing a direct enzymatic role in ligation of WTA to peptidoglycan substrates (Schaefer et al., 2017). Thus, it is likely that the LytR may function as a ligase in the retention of both CPS and TAs to the cell wall in *S. pneumoniae*.

Knocking down Cps2A did not impair the growth profile in *S. pneumoniae* D39 (Eberhardt et al., 2012), suggesting that the loss of CPS alone may not influence the division or autolysis and hence the growth rate. This study demonstrated that the *lytR* mutant grew very slowly and the stationary phase was missing (**Figure 2**). On the basis of the growth results, the phenotypes of *lytR* mutant are consistent with the typical growth defect caused by repression of genes in TA biosynthesis (Liu et al., 2017). This may be explained by the observation that *lytR* was involved in the biosynthesis of TAs (**Figure 6**). It is known that TA was important for the biological roles in *S. pneumoniae*; however, the attachment to the cell remains elusive. Our previous results suggested that *rafX* may function as a ligase attaching the TA precursors to the cell wall; However, *rafX* inactivation did not lead to complete loss of the TAs in *S. pneumoniae* strains (Wu et al., 2014), indicating the involvement of other enzymes in this process. In this study, deletion of the *lytR* gene also impaired the attachment of TAs to bacteria, suggesting the enzymatic effect of LytR in this process. Of note, the size of TAs was different in the *lytR* mutant compared to wild-type and the complemented strains (**Figure 6**). One possible explanation for this may be relevant to the less amounts of TAs to cell wall which may lead to faster migration of TAs in the *lytR* mutant than that of the wild-type strain. Another possible reason is that LytR might lead to modification in TAs which needs to be further investigated. Nevertheless, our findings strengthen our knowledge on pneumococcal TA biosynthesis.

Contrary with previous studies showing that LytR could not be inactivated alone in *S. pneumoniae* D39 (Eberhardt et al., 2012), the present study replaced the *lytR* gene with *erm* cassette. The deletion of *lytR* gene was proved to be a nonpolar event verified by reverse transcription (RT)-PCR and sequencing. In addition, the D39Δ*lytR* mutant did not display very obvious morphological and division abnormity as did the R6Δ*lytR* mutant (Johnsborg and Havarstein, 2009). The discrepancy in obtaining the *lytR* mutant may be due to the different genetic background pneumococcal strains used, or may be caused by the difference in selection marker where kanamycin was used as a selection marker for gene deletion in their study.

In this study, we observed an increased amount of CPS in the culture supernatant of the *lytR* mutant, which was an indication of unbound portions due to the function loss of LytR. This is true because exogenous addition of LytR reduced the amount of CPS in culture supernatant and more CPS was observed in bacterial cells (**Figure 5**). It was found that the complemented strain (ectopically complemented with the plasmid *lytR*-pJWV25) produced more CPS than did the wild-type D39 as shown in **Figure 4**. We therefore examined the level of LytR in the complemented strain and found that a higher level of LytR was produced in complemented strain than the wild-type D39 (Supplementary Figure S3). This observation highlights that the ligation of CPS to the cell wall may be dependent on the LytR concentrations, which is consistent with the enzyme reaction feature.

It has been reported that the inactivation of *cps2A* alone only led to 29% reduction in the amount of surface CPS. In this study, it was found that about half of the capsule was missing on the surface of bacterial cells in the *lytR* mutant (**Figures 4**, **5**). It was surprising that the *lytR* deficient mutant had equal amount of CPS as the *cpsA lytR* double mutant shown in a previous study (Eberhardt et al., 2012). It seems that LytR was more important than Cps2A in the retention of CPS to the cell wall.

It is of note that despite we named Δ*lytR*-C as the complemented strain, as shown in **Figures 6**, **7**, the phenotype of Δ*lytR*-C was not restored to the wild-type levels. This is because that the complemented effect relays on the concentrations of Zn^2+^ (Eberhardt et al., 2009). Thus, when complemented strain was inoculated into the animals, the concentrations of Zn^2+^ may be not sufficient to support a continuous expression of LytR and hence may lead to the attenuation of the complemented strain, and this may be used to explain why complemented strain was not as virulent as the wild-type in the infection experiments. Although the Δ*lytR*-C strain failed to kill animals as the wild type D39 in animal infection experiments, the results in **Figure 7** did not show significant difference in resistance to killing by phagocytosis among wild type D39, D39Δ*lytR*-C and D39Δ*lytR*+ ΔTM-LytR suggesting the complementation of LytR and the contribution of LytR in bacterial virulence.

## Conclusion

Our results provide strong evidence that pneumoccal LyR is critical for some basic cellular functions and important for pneumococcal virulence. Since LytR is a membrane protein and is surface exposed, we anticipate that pneumococcal LytR can be a novel drug target to cure pneumococcal infection.

## Author Contributions

YY, XZ, and KW conceived and designed the experiments. WY, JZ, and ZS performed the experiments. WY, YY, XZ, and KW analyzed the data. YY, XZ, and KW contributed the reagents, materials, and analysis tools. WY, XZ, and KW wrote the paper. All authors read and approved the final manuscript.

## Conflict of Interest Statement

The authors declare that the research was conducted in the absence of any commercial or financial relationships that could be construed as a potential conflict of interest.
