# Supplementary material for: Pneumococcal LytR Protein Is Required for the Surface Attachment of Both Capsular Polysaccharide and Teichoic Acids: Essential for Pneumococcal Virulence
Source: Front Microbiol. 2018 Jun 13;9:1199. doi: 10.3389/fmicb.2018.01199 (PMC6008509; doi:10.3389/fmicb.2018.01199)
Supplement: Supplementary file 1 [file Data_Sheet_1.doc]

TABLE 1 | Bacterial strains and plasmids used in this study.

| **Strain/plasmid** | | **Relevant properties** | **Antibiotic concentrations** | | **Source or references** | |
| --- | --- | --- | --- | --- | --- | --- |
| ***STREPTOCOCCUS PNEUMONIAE*** | |  |  | |  | |
| D39 | |  |  | | NCTC | |
| D39Δ*lytR* (*SPD_1741*) | | Erm^R^ | 0.25 μg/ml | | This study | |
| D39Δ*lytR* pJWV25-lytR | | pJWV25,SPD_1741,Erm^R^,tet^R^ | Erm: 0.25μg/ml; Tet: 0.25 μg/ml | | This study | |
| R6 | |  |  | | ATCC | |
| R6Δ*lytR* (*SPD_1741*) | | Erm^R^ | 0.25 μg/ml | | This study | |
| R6 Δ*lytR* pJWV25-lytR | | pJWV25, SPD_1741, Erm^R^, tet^R^ | Erm: 0.25μg/ml; Tet: 0.25 μg/ml | | This study | |
| ***ESCHERICHIA COLI*** | |  |  | |  | |
| *E. coli* DH5α | | Cloning strain |  | | Takara | |
| *E. coli* BL21(DE3) | | Expression strain |  | | Takara | |
| ***PLASMIDS*** | |  |  | |  | |
| pJWV25 | | Zn^2+^-dependent production; Tet^R^ | Tet:12.5 μg/ml (in *E. coli*) | | Eberhardt *et al.*, 2009 | |
| pET-28a (+) | | Kana^R^, (over-expression vector) | 50 μg/ml (in *E. coli*) | | Takara | |
| pJWV25-lytR | | pJWV25, SPD_1741, tetR | Tet: 2.5 μg/ml | | This study | |
| **TABLE 2 | Primers used in this study.** | | | | | | |
| **Primer** | **Sequence (5′-3′ )** | | | **Size (bp)** | **Description** |  |
| lytR |  | | |  |  |  |
| lytR-F | CGGGATCCATGGTTAAAAAAATTATTGGAATGG | | | 924 | ΔTM-LytR |  |
| LytR-R | CCGCTCGAGTTAATTATCTTCATCACCAACAGGT | | |  |  |  |
| ΔlytR |  | | |  |  |  |
| ΔlytR-P1 | AAAGTCCCACCTATACTATCGTGAG | | |  |  |  |
| ΔlytR-P2 | ATCAAACAAATTTTGGGCCCGGTTCTACTAACCTATCAGTTTACCCA | | |  |  |  |
| ΔlytR-P3 | ATTCTATGAGTCGCTGCCGACTAATAAAAAAATCAATCGTAGGAAAA | | |  |  |  |
| ΔlytR-P4 | ATAGCAAAAGTCTCGTAAAGAAATT | | |  |  |  |
| pJWV25-lytR |  | | |  |  |  |
| pJWV25-lytR-F | GGACTAGTATGGTTAAAAAAATTATTGGAATGG | | | 1517 | *Spe* I site |  |
| pJWV25-lytR-R | CGGACTAGTATGGTTAAAAAAATTATTGGA | | |  | *Not* I site |  |
| Erm |  | | |  |  |  |
| Erm-F | CCGGGCCCAAAATTTGTTTGAT | | | 780 | *Erm* resistance marker |  |
| Erm-R | AGTCGGCAGCTCATAGAAT | | |  |  |  |
| Tet2 |  | | |  |  |  |
| Tet2-F | CCGGGCCCAAAATTTGTTTGAT | | |  |  |  |
| Tet2-R | TCCCAAAGTTGATCCCTTAACGA | | |  |  |  |
| GFP |  | | | 165 | *Tet* resistance marker |  |
| gfp-F | AAAGGAGAAGAACTTTTCACTGGAG | | |  |  |  |
| gfp-R | AGTAGTGACAAGTGTTGGCCATGGA | | |  |  |  |
| lytA- [promoter](javascript:void(0);)01 | CTGTAAAAATATACTTTTGAAAAG (5’-labeled with biotin) | | |  |  |  |
| lytA- [promoter](javascript:void(0);)02 | ATTCTACTCCTTATCAATTAAAAC | | |  |  |  |
| rafX-[promoter](javascript:void(0);)01 | AGCCTTGATATGGTGGATAAAATAG(5’ -labeled with biotin) | | |  |  |  |
| rafX-[promoter](javascript:void(0);)02 | TAATTCCCAATAAAATCAGCTCTTT | | |  |  |  |
| cps-[promoter](javascript:void(0);)01 | TACACATCGCTTCTAAAATATTGT(5’ -labeled with biotin) | | |  |  |  |
| cps-[promoter](javascript:void(0);)01 | TTAAAACGTCTACTCATGATTAACA | | |  |  |  |


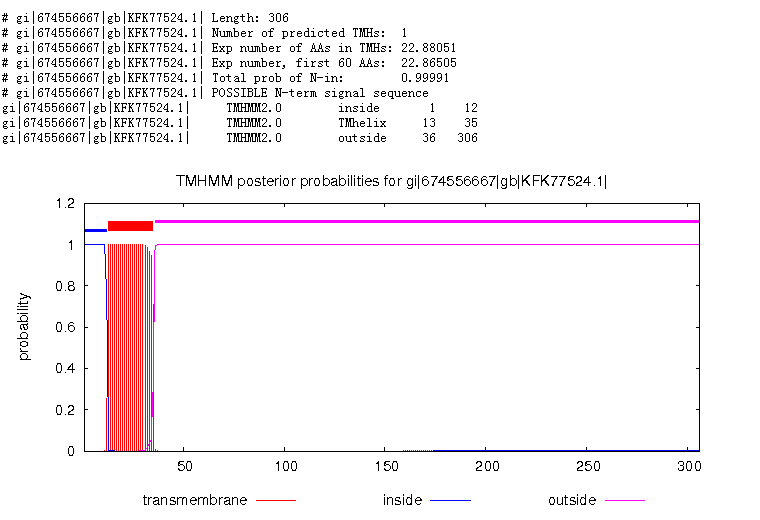


Fig. S1. Prediction of trans-membrane structure of *Streptococcus pneumoniae* R6 LytR using TMHMM.


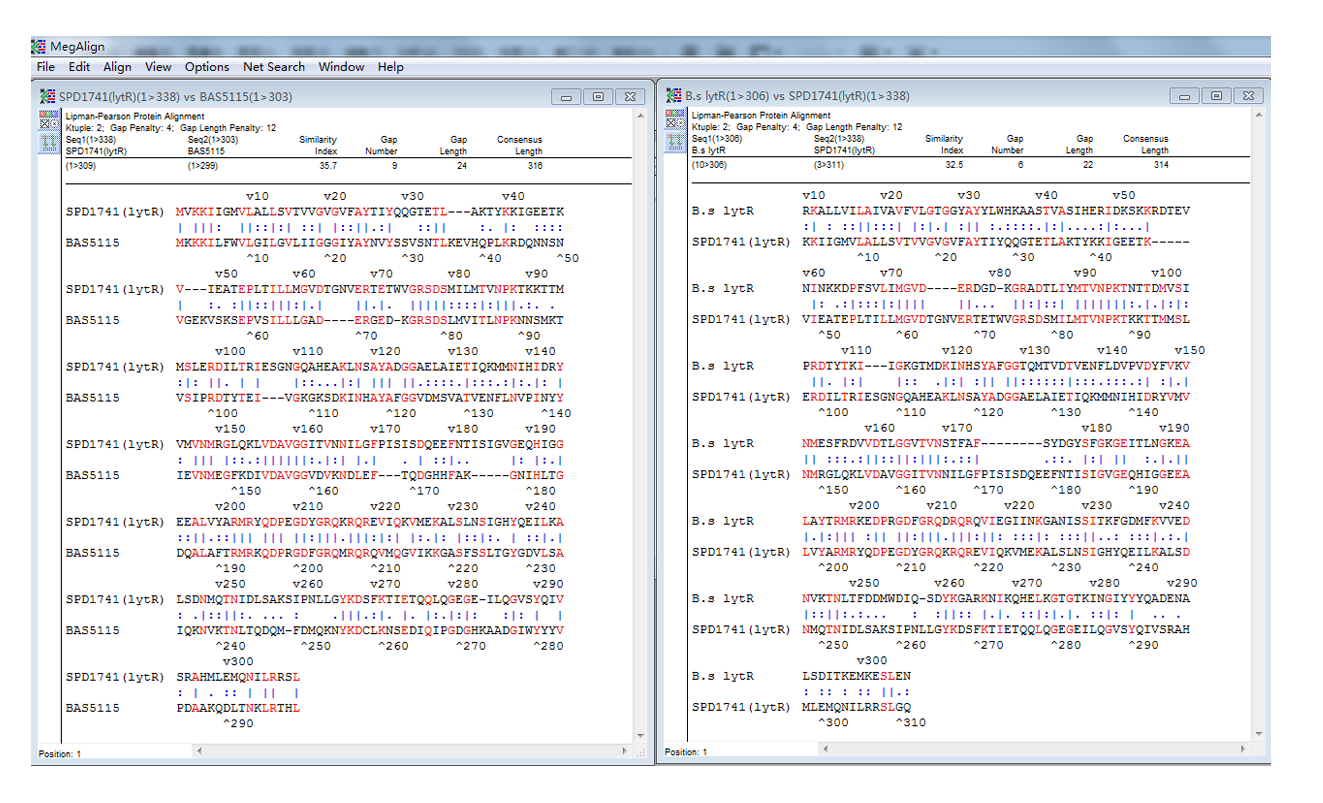


Fig. S2. Sequence alignment between *B. subtilis* DJ97_1211 and *S. pneumoniae* SPD_1741.

*S. pneumoniae* LytR (SPD_1741) showed 32.5% identify with the *B. subtilis* DJ97_1211 in full-length amino acid sequence


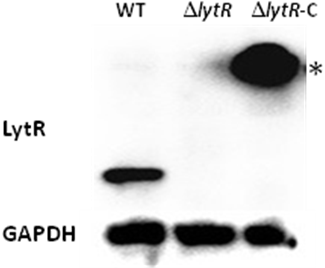


Fig. S3. Compared to the wild type strain, the LytR was expressed in larger amount following inducing with 0.15 mM Zn^2+^. * denotes the fusion protein of GFP-LytR which was greater in molecular weight than LytR.
